# Archipelago Method for Variant Set Association Test Statistics

**DOI:** 10.1002/gepi.70025

**Published:** 2026-01-06

**Authors:** Dylan Lawless, Ali Saadat, Mariam Ait Oumelloul, Luregn J. Schlapbach, Jacques Fellay

**Affiliations:** ^1^ Department of Intensive Care and Neonatology University Children's Hospital Zürich, University of Zürich Zürich Switzerland; ^2^ GlobalHealth Institute, School of Life Sciences ÉcolePolytechnique Fédérale de Lausanne Lausanne Switzerland

**Keywords:** Archipelago, combined, GWAS, RVAT, VSAT

## Abstract

Variant set association tests (VSAT), especially those incorporating rare variants via variant collapse, are invaluable in genetic studies. However, unlike Manhattan plots for single‐variant tests, VSAT statistics lack intrinsic genomic coordinates, hindering visual interpretation. To overcome this, we developed the Archipelago method, which assigns a meaningful genomic coordinate to VSAT P values so that both set‐level and individual variant associations can be visualised together. This results in an intuitive and information rich illustration akin to an Archipelago of clustered islands, enhancing the understanding of both collective and individual impacts of variants. We conducted three validation studies spanning simulated and real datasets across small and biobank‐scale cohorts, from 504 individuals up to 490,640 UK Biobank participants. We integrated single‐variant genome‐wide association studies (GWAS) with gene‐ and protein pathway‐level rare‐variant collapse. These studies included the 1KG GWAS cohort, the Pan‐UK Biobank GWAS with DeepRVAT WES gene‐level study, and the UKBB WGS gene‐level UTR collapsing PheWAS. The Archipelago plot is applicable in any genetic association study that uses variant collapse to evaluate both individual variants and variant sets, and its customisability facilitates clear communication of complex genetic data. By integrating at least two dimensions of genetic data into a single visualisation, VSAT results can be easily read and aid in identification of potential causal variants in variant sets such as protein pathways.

## 1 Introduction

Variant set association tests (VSATs) are methods in which groups of variants are collapsed and analysed jointly to enhance an association signal. VSAT, particularly those incorporating rare variants (e.g. minor allele frequencies below 1%), have become indispensable in genetic association studies (Derkach et al. 2014; Lee et al. 2014; Larson et al. 2019; Povysil et al. 2019; Li et al. 2022; Clarke and Holtkamp 2024). Next‐generation sequencing has enabled the comprehensive detection of rare variants, thereby complementing traditional single‐variant tests (Nicolae 2016; The UK Biobank Whole‐Genome Sequencing Consortium et al. 2025). While Manhattan plots effectively visualise single‐variant associations (Turner 2018), a comparable graphical representation for VSAT results has been lacking.

To name a small few, VSAT methods include burden tests (e.g. CAST (Morgenthaler and Thilly 2007), CMC (Li and Leal 2008), weighted‐sum (Madsen and Browning 2009)), adaptive burden tests (e.g. alpha‐sum (Han and Pan 2010), VT (Price et al. 2010), KBAC (Liu and Leal 2010)) and variance component tests. Variance component approaches, such as the C(α) test (Neale et al. 2011; Neyman 1979) and kernel machine regression methods (Larson et al. 2019), offer flexible strategies for assessing the combined effect of rare variants. In particular, the sequence kernel association test (SKAT) (Wu et al. 2011) and its extensions including SKAT‐O (Lee et al. 2012b), MSKAT (Lee et al. 2012a), famSKAT, and RC‐SKAT (Chen et al. 2013; Ionita‐Laza et al. 2013a, b; Dutta et al. 2019) have become widely adopted.

More recently, the variant‐set test for association using annotation information (STAAR) framework (Li et al. 2020, 2022) has been introduced, utilising the aggregated Cauchy association test (ACAT) (Liu et al. 2019) to combine multiple annotation‐based P values for each variant. Despite these methodological advances, the lack of a natural genomic coordinate for VSAT P values hinders their visual interpretation.

To address this gap, we introduce the Archipelago plot. By assigning a genomic coordinate to the VSAT P value based on the average of its constituent variants, this method facilitates the simultaneous visualisation of aggregated and individual variant effects. Notably, multiple types of genome‐wide association study (GWAS), VSAT, and rare variant association test (RVAT), including SKAT‐O and ACAT, have been highly successful and scalable to national biobank cohorts where this method can be applied. Examples include a Pan‐UK Biobank (UKBB) GWAS (Karczewski et al. 2024) of 7,266 phenotypes (~441k individuals), DeepRVAT gene‐level rare variant WES analyses (~470k individuals) (Clarke et al. 2024), the UKBB WGS UTR collapsing PheWAS (~490k individuals) (The UK Biobank Whole‐Genome Sequencing Consortium et al. 2025). Meta‐analysis of TOPMed whole‐genome sequencing data and UKBB whole‐exome sequencing data, encompassing ~200k individuals, has demonstrated the utility of these methods (Li et al. 2023). Similarly, rare non‐coding variation in complex human phenotypes has been investigated on ~333k individuals from three cohorts: UK Biobank (N = ~200k), TOPMed (N = ~88k) and All of Us (N = ~45k) (Hawkes et al. 2024). Given their proven success, VSAT methods are likely to remain essential in future studies, and our Archipelago plot is designed to be applicable to such investigations.

## 2 Implementation

**Figure 1:**
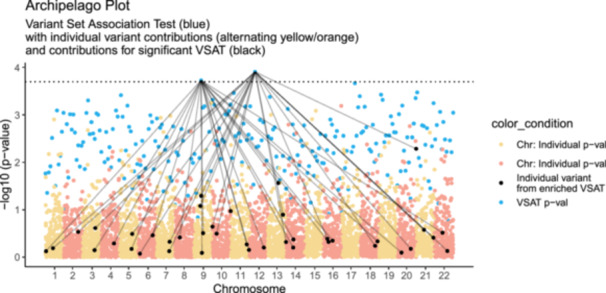
Synthetic data for human autosomal chromosomes 1–22. Contains 250 variant sets with 5000 individual variants. Each significantly enriched VSAT P value (blue) is mapped to its constituent individual P values (yellow and orange points). Significance threshold based on the number of VSATs.

The Archipelago plot is a novel visualisation technique developed to facilitate the interpretation of both (1) variant‐set association testing (VSAT) P values (2) single variant P values in the context of the variant set. Its design is based on the commonly used Manhattan plot but accounts for the unique properties of VSAT statistics. Figures 1 uses synthetic data to illustrate output that might be expected in RVAT for case‐control analysis. Briefly, 5,000 independent variants were simulated for GWAS results and 250 VSAT tests (20 variants per set). VSAT P values were drawn from a log‐normal distribution using a minimal threshold (0.05/10), while GWAS P values were uniformly sampled between 0.05/100 and 1, with base pair positions and chromosome numbers assigned at random. This represented 250 VSAT P values and 5000 independent variants. As shown in Figure 1, blue points indicate the joint VSAT P value as produced by SKAT‐O or other statistical methods. Alternating yellow and orange points indicate the individual P values from each variant as produced by a regression or some other statistical method (e.g. R package SKAT: $param$p.val.each, P value for each single variant in a set‐based test, or as calculated in single variant analysis). Alternating colours clarify the chromosome position. Lines connect the VSAT P value (blue) to its constituent individual variants to indicate variant set grouping. These edges are only shown for variants which are also in the significantly enriched VSAT P value group.

The precise protocol summary, definition, and algorithm can be found in Sections 13.7–13.9. User settings of the R package can be read in Section 13.6. The R code used in this manuscript can be found in the GitHub package repository. The main steps of the process are:

1. Within each variant set, get the average genome‐wide genomic coordinate and assign it to the VSAT group P value.
2. Optionally, normalise the VSAT x‐axis distribution for dispersion to prevent centre clustering in dense datasets. Ranking of VSAT P values also provides priority in the event of overlaps.
3. Optionally, map the VSAT position to each individual variant from the set.

**Figure 2:**
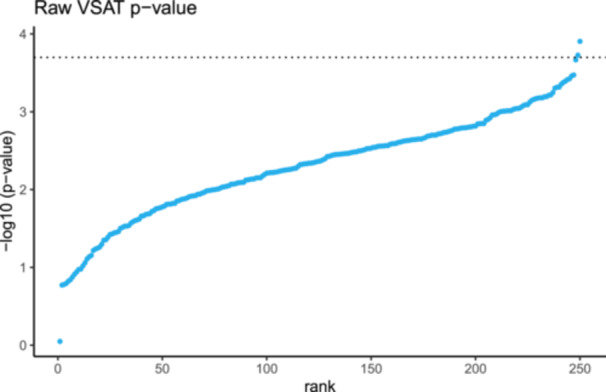
Raw VSAT P values have no natural x‐axis position and must be ranked on association strength or some arbitrary ranking. The same VSAT result shown in Figure 1 (blue) is shown here without the use of the Archipelago method.

Since variant sets (such as protein pathways) have no specific single genomic coordinate, it is otherwise difficult to assign a logical x‐axis position. Figure 2 illustrates the default format of raw VSAT P values, which will either be ranked by their P value strength or some other rank based on the variant set construction which results in an arbitrary x‐axis distribution. The Archipelago plot assigns these same VSAT results into their x‐axis location as the ranked average of the individual variant set genomic coordinate, thus representing an information‐rich illustration. For example, a variant set that is made from mostly chromosome 1 variants, will appear on the left, near its constituent variant P value points from chromosome 1. Genome‐wide variant sets will appear near the centre, however to prevent strong clustering in very dense datasets we also provide the method for ranked dispersion based on genomic coordinates for clearer illustration of the VSAT P value.

To ensure that VSAT points do not cluster and heavily overlap, ranking is applied before finding the variant set average based on the VSAT P value. That is, the x‐axis position of the strongest association will have priority. Although overlap is unlikely in genome‐wide testing, it may occur where variant sets are constructed from a narrow set of genomic locations. In all figures a significant P value threshold was used which would typically be derived based on the number of independent tests, such as the number of variant sets. In these examples we used the arbitrarily chosen $P\;\;\text{value}\;=\frac{.05}{250}=.0002$ indicating 250 variant set tests.

We include a set of Supporting Figures S2a, S2b, and S2c which contain the same dataset as Figure 1. However, each version illustrates the decreasing levels of clarification information to demonstrate the layers of annotation. Figure S2a shows the original plot without the figure legend. Figure S2b drops the edge highlights for the significantly enriched VSAT to reveal all connections, which can be difficult to read in high density plots. Figure S2c next drops the individual variant P value highlighting. Conversely, Figure S3 adds an additional layer of information by adding two colours for the significantly enriched variant sets, which is useful when there are multiple enriched variant sets but otherwise may be distracting. We demonstrate a set of sparse plots in Figures S4a, S4b, and S4c. These smaller dataset examples use synthetic data to represent 500 qualifying variants, and VSAT of 20 genes/variants per variant set (25 VSAT P values).

Customisation settings are described in Section 13.10. Figure S5 shows an example of customised colours. Figure S6 shows all customisable elements (title, subtitle, colours, colour labels, critical threshold line, genomic coordinate, show title and subtitle, and show legend). Figures S7–S8 shows all 16 colour themes. The R package logo is shown in Figure S9.

## 3 Expansion With Additional Information Layers

The variant‐set test for association using annotation information (STAAR) method has special features since each individual variant P value (pSTAAR) is itself made from an aggregated P value derived from the set of annotation weighted P value using the ACAT method (Li et al. 2020, 2022; Liu et al. 2019). Therefore, a third level of information hierarchy is present. In the basic Archipelago plot we have two layers: (1) VSAT P value and (2) individual P value. The third pSTAAR level might be illustrated in a sub‐plot which focuses on the significantly enriched VSAT group. This sub‐plot is then free to show x‐axis genomic coordinates, and y‐axis P value which is used for the Cauchy distribution aggregation of $f\left(p_{n}\right)$.

**Figure 3:**
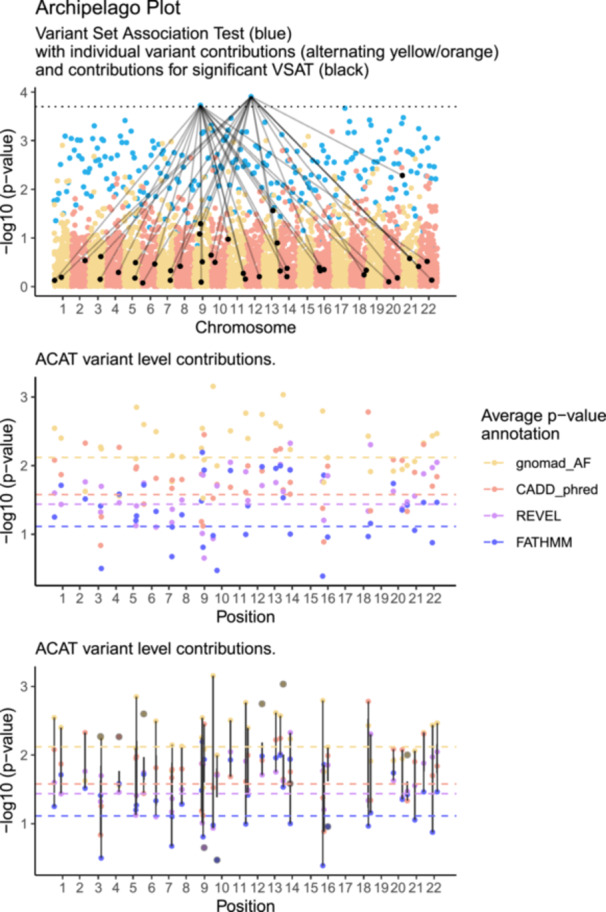
Panel [1] Archipelago plot as per Figure 1 with two significantly enriched variant sets (blue), 40 contributing individual variants (black). Panel [2] ACAT P values based on 4 annotation layers for each one of the individual (black) variants. Horizontal lines show the average annotation P value as an estimate of the hierarchy of contribution. Panel [3] overlays line/group box to show the spread of ACAT P values for each single variant.

To demonstrate the source of annotation based ACAT P values, Figure 3 panel [1] shows the Archipelago plot with two significantly enriched variant sets (blue), 40 contributing individual variants (black). Panel [2] then shows the ACAT P values for 4 annotation layers for each one of the individual (black) variants. To clarify that each single variant position will have four annotation P values, panel [3] overlays line/group box to show the spread of ACAT P values for each single variant. We note that highly correlated annotation layers are likely since many prediction tools rely on population genetics frequency as an indicator of variant effect quantification. Therefore, it would be reasonable to also show a further plot of the correlation between the annotation layers. While this is a simplified and specific example, we imagine that other similar applications will exist for VSAT with third layers of information that consist of x‐axis genomic coordinate and y‐axis test statistic.

## 4 Validation

We validated the Archipelago method across three distinct scenarios, spanning both simulated and real datasets of single‐variant GWAS, and gene‐ or pathway‐level collapse VSAT/RVAT:

1. **1000 Genomes (1KG)**—504 East Asian samples with a simulated binary trait GWAS and a pathway‐level VSAT (Fairley et al. 2020).
2. **Pan‐UK Biobank (UKBB)**—469,382 UKBB samples with a quantitative trait GWAS and DeepRVAT gene‐level WES RVAT (Clarke et al. 2024; Karczewski et al. 2024).
3. **UKBB WGS UTR PheWAS**—490,640 UKBB samples with GWAS and untranslated region (UTR) collapsing gene‐level WGS RVAT (The UK Biobank Whole‐Genome Sequencing Consortium et al. 2025).

These settings demonstrate Archipelago's versatility in unifying association signals across variant resolution and trait architecture.

### 4.1 Validation in 1KG Using GWAS and Simulated VSAT

**Figure 4:**
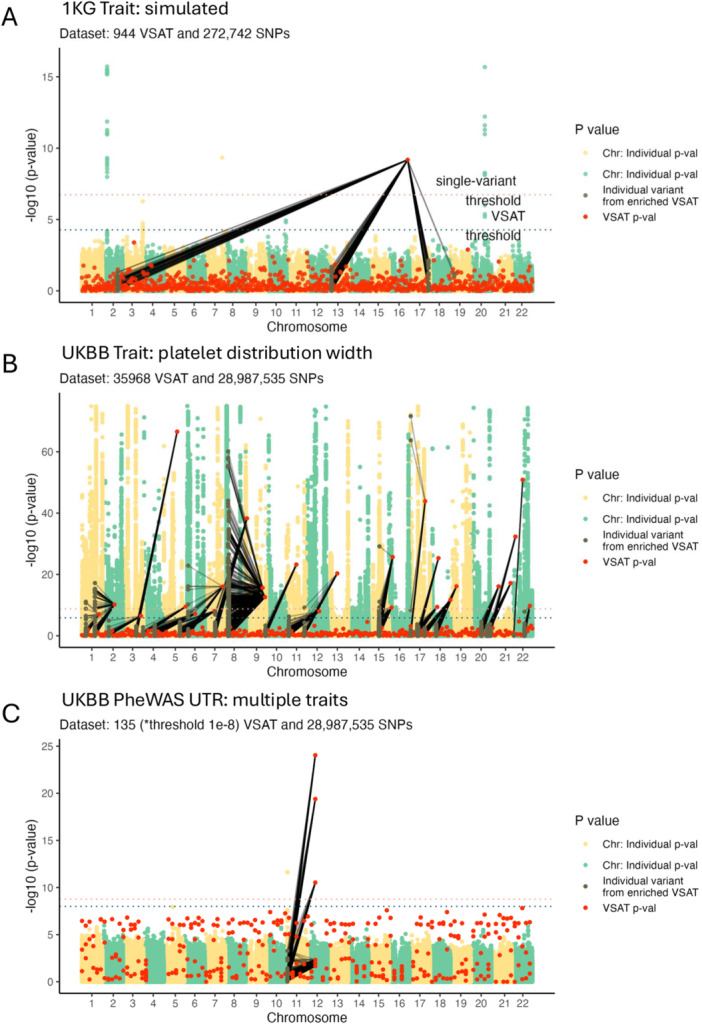
Archipelago plots across three validation settings. (A): 1000 Genomes East Asian cohort (n=504) with simulated pathway‐level binary trait (Fairley et al. 2020). (B): Pan‐UK Biobank (n=469,382) platelet distribution width trait using WES GWAS and DeepRVAT gene‐level VSAT (data trimmed at 1e‐75 for distracting outliers) (Clarke et al. 2024; Karczewski et al. 2024). (C): UKBB WGS UTR collapsing PheWAS (n=490,640) using WGS GWAS and UTR gene‐level VSAT (*The UK Biobank Whole‐Genome Sequencing Consortium et al. 2025 defined the significance threshold for their PheWAS dataset) (The UK Biobank Whole‐Genome Sequencing Consortium et al. 2025). In all panels, Archipelago visualises variant‐level and set‐level associations across the genome, enabling joint interpretation of individual and collapsed variant signals.

We validated the Archipelago method by first reproducing a GWAS study (Supporting, Section 13) using the public dataset of 504 East Asian individuals from the 1000 Genomes Project phase 3 (version 5, hg19) (Fairley et al. 2020). Since 1KG data lack a common disease phenotype, we simulated a binary trait (250 cases, 254 controls) under a heritability of 0.8, then applied standard QC steps (e.g. splitting multi‐allelic variants, normalising genotypes, LD pruning, KING‐based relatedness checks) to retain 500 samples and 1,224,104 single nucleotide polymorphisms (SNPs). A single‐variant GWAS was run via logistic regression with Firth correction, and a VSAT was conducted using protein pathways from ProteoMCLustR (941 pathways) and SKAT‐O. Tables S1–S2 illustrate the resulting style of pathway‐ and SNP‐level outputs required; the Archipelago plot (Figure 4A) integrates both in a single genomic view.

The enriched variant signals displayed in Figure 4A were assessed based on their allele frequency and displayed in Figure S1. In this dataset, top GWAS signals involved highly common variants (n = 67, mean allele frequency 0.333, min = 0.13, max = 0.49), whereas the top VSAT signal (set_ID 532) included 261 variants with a mean frequency of 0.151 (min = 0.01, max = 0.48), reflecting a different genetic architecture captured by pathway‐based collapse.

### 4.2 Validation in Pan‐UK Biobank Using GWAS and Gene‐Level DeepRVAT

We selected a notable quantitative trait “platelet distribution width” for validation based on the large UKBB rare variant WES study (minor allele frequency <0.1%) for DeepRVAT by Clarke et al. (2024). We performed the analysis using the two complementary layers: a Pan‐UK Biobank SNP‐level GWAS (Karczewski et al. 2024) and gene‐level rare variant association results from DeepRVAT on UK Biobank WES (469,382 participants) (Clarke et al. 2024). Together these layers comprise 29 million SNP‐level GWAS statistics and 35,968 gene‐level RVAT tests. The joint analysis result of Archipelago is shown in Figure 4B.

For this highly polygenic and heritable trait, the Pan‐UKBB GWAS identified hundreds of significant loci, including multiple regions with $P<10^{-50}$, consistent with known biology of platelet regulation. DeepRVAT identified 26 gene‐level associations, capturing additional rare variant signals not resolved at the common single‐variant level. The integrated view enabled systematic comparison of RVAT and GWAS signals, capturing convergence and divergence between rare and common variant associations. Eight genes (*APOA5*, *MOB3C*, *DOCK5*, *LPL*, *GP1BA*, *PEAR1*, *MPIG6B*, *PLEKHO2*) showed significant association in both GWAS and RVAT layers, revealing a novel insight.

### 4.3 Validation in UKBB WGS Using GWAS and UTR Collapsing PheWAS

We used the flagship UK Biobank whole‐genome sequencing study of 490,640 participants as a large‐scale, well characterised validation resource (The UK Biobank Whole‐Genome Sequencing Consortium et al. 2025). Within this study, we selected the UTR gene‐collapsing PheWAS as a notable analysis for integrating rare non‐coding burden with GWAS, given its cross‐ancestry design, deep phenotyping, and improved coverage of UTR and structural variation relative to whole exome sequencing (WES). A total of 135 traits were tested (e.g. ICD‐10 D56 thalassaemia and ICD‐10 I50 heart failure). The study reported a single genome‐wide significant binary association in the UTR analysis, thalassaemia (ICD‐10 D56), localising to *HBB* under the $5^{\prime }+3^{\prime }$ UTR model at $P=9.16\times 10^{-25}$ with an empirically validated threshold of $P\leq 1\times 10^{-8}$ (figure 4 of The UK Biobank Whole‐Genome Sequencing Consortium et al. 2025).

Using Archipelago, in Figure 4C we reproduced *HBB* as the top binary UTR association and showed that this rare UTR burden maps back to individual *HBB* SNPs in the Pan‐UKBB GWAS (Karczewski et al. 2024). Separately, the GWAS layer also exhibited a significant peak (yellow chromosome 11 SNPs) across the beta globin cluster on chr11p15.5, where the lead signal overlaps nearby genes including *HBE1*, *HBG2* and *OR51B5*, consistent with local linkage disequilibrium, while the UTR collapsing result attributes the gene‐level effect to *HBB*. This validation provides a simple clinically interpretable positive control. Archipelago can thus illustrate set‐level rare UTR burden to constituent GWAS variants and provide a novel insight using both layers.

## 5 Discussion

We have introduced the Archipelago plot as a novel method for interpreting VSAT results alongside individual variant P values, as produced from SKAT‐O or some other statistical method after variant collapse (Derkach et al. 2014; Lee et al. 2014; Povysil et al. 2019). In contrast to traditional Manhattan plots that are restricted to single‐variant associations with clear genomic coordinates, the Archipelago plot assigns a representative genomic location to the VSAT P value based on the average of its constituent variants (Turner 2018). This enables a direct comparison of the aggregated signal with individual variant effects within a familiar framework.

The absence of a natural genomic coordinate for VSAT P values has previously hindered the integrated visual interpretation of variant set and single‐variant results. By linking the VSAT P value to the genomic positions of the individual variants, the Archipelago plot offers an intuitive depiction of the relationship between variant set effects and the underlying individual signals. This approach may assist in identifying potential causal variants or pathways and can be readily extended to accommodate additional layers of information, such as annotation‐based P values derived using methods like ACAT. We have seen that this method could be applied to national biobank scale genomic studies with relative ease (Li et al. 2023; Hawkes et al. 2024).

While the method was developed with rare‐variant testing in mind, its customisable design permits application to any genetic association study that utilises variant collapse. Overall, the Archipelago plot provides a modest yet useful tool for the clear communication of complex genetic data, contributing to improved interpretability in large‐scale VSAT analyses.

## Author Contributions

Dylan Lawless designed and wrote the work and performed analysis. Ali Saadat and Mariam Ait Oumelloul wrote the work. Luregn J. Schlapbach and Jacques Fellay led project management and funding.

## Ethics Statement

This study only used data which was previously published and publicly available, as cited in the manuscript. This SwissPedHealth study, under which this work was carried out, was approved based on the advice of the ethics committee of Northwest and Central Switzerland (EKNZ, AO_2022‐00018). The study was conducted in accordance with the Declaration of Helsinki.

## Conflicts of Interest

The authors declared no conflicts of interest.

## Supporting information

Supplementary Information

## Data Availability

This study is reproducible using the provided code repository https://github.com/DylanLawless/archipelago. The data repository additionally provides the validation studies data (1.5G), which are also reproducibly self‐contained (Lawless 2025) https://doi.org/10.5281/zenodo.16880622. Detailed data sources and automated download links are provided within each script of the online data repository for the 1KG, Pan‐UKBB, and DeepRVAT studies. The original sources are: The 1000 Genomes Project phase 3 version 5 (1KG 3v5), genome build human_g1k_v37. fasta (hg19) (Fairley et al. 2020) http://ftp.1000genomes.ebi.ac.uk/vol1/ftp/release/20130502/. Pan‐UK Biobank GWAS summary statistics: the UKBB GWAS (continuous‐30110‐both_sexes‐irnt. tsv. bgz) and phenotype manifest (Karczewski et al. 2024) https://pan.ukbb.broadinstitute.org. DeepRVAT gene‐trait association testing results on the 470k UK Biobank WES dataset (UKBB_470k_deeprvat_results. csv) (Clarke et al. 2024; Clarke and Holtkamp 2024) https://doi.org/10.5281/zenodo.12736824. UKBB WGS UTR collapsing PheWAS results in 490k: Supplementary Table 16 from (The UK Biobank Whole‐Genome Sequencing Consortium et al. 2025) (Significant and suggestive gene‐phenotype associations identified in the UTR PheWAS collapsing analysis across $5^{\prime }$ UTR, $3^{\prime }$ UTR, $5^{\prime }+3^{\prime }$ UTR and coding sequence + $5^{\prime }+3^{\prime }$ UTR) https://static-content.springer.com/esm/art%3A10.1038%2Fs41586-025-09272-9/MediaObjects/41586_2025_9272_MOESM10_ESM.xlsx.
