# Supplementary material for: Archipelago Method for Variant Set Association Test Statistics
Source: Genet Epidemiol. 2026 Jan 6;50(1):e70025. doi: 10.1002/gepi.70025 (PMC12771271; doi:10.1002/gepi.70025)
Supplement: Supplementary file 1 — Supplementary Information [file GEPI-50-0-s001.pdf]

Click to BUY NOW! PDF-XChange Editor www.pdf-xchange.com
Click to BUY NOW! PDF-XChange Editor www.pdf-xchange.com

13 Supplemental

13.1 Input

Tables S1 and S2 demonstrate the minimal input format required by Archipelago, showing the first few lines from validation study 1 (**section** 4.1):

1. The VSAT table (**Table** S1), typical of SKAT, contains one row per tested variant set (e.g. gene or pathway), identified by `set_ID` and associated P value. As VSAT results are aggregated across multiple variants, no genomic position is provided.

2. The GWAS table (**Table** S2), typical of Plink, contains single-variant results, with genomic coordinates (`CHR` and `BP`) and a P value for each SNP. To enable integration, each SNP must also be annotated with a `set_ID`, matching the grouping used in the VSAT layer.

This shared `set_ID` acts as a join key across resolution layers, allowing Archipelago to combine variant associations with aggregated signals from gene, pathway, or other set-based tests. To align SNP-level set-level results, a shared common key might by missing especially from independent studies. In these cases we used R biomaRt to map the consensus feature based on Ensembl GRCh38 genomic coordinates.

set_ID	P value
1	0.7557
2	0.2883
3	0.5410
4	0.7858

Table S1: **VSAT results from SKAT-O.** Each variant set (i.e. protein pathway) requires an index by `set_ID` resulting in a single P value per association test (source: validation study 1KG `df_pathway_sim.csv`).

Click to BUY NOW! PDF-XChange Editor www.pdf-xchange.com
Click to BUY NOW! PDF-XChange Editor www.pdf-xchange.com

set_ID	CHR	BP (position)	P value
1	1	160289049	0.2560
1	1	160295829	0.5597
1	1	160300635	0.9210

Table S2: **GWAS results from Plink.** In GWAS, each variant is reported with its chromosome (`CHR`), base-pair position (`BP`), and association P value. To enable integration with the VSAT results in Table S1, each SNP is annotated with its corresponding `set_ID`, derived by mapping to GRCh38 genes grouped into protein pathways. (source: validation study 1KG `df_snp_id.csv`).

13.2 Validation methods

13.2.1 Validation method in 1KG GWAS and simulated gene-level VSAT

We performed a validation test using a popular reference dataset consisting of 504 EAS individuals from 1000 Genomes Project phase 3 version 5 (1KG 3v5), genome build human_g1k_v37.fasta (hg19) (http://ftp.1000genomes.ebi.ac.uk/vol1/ftp/release/20130502/) (31). This dataset contained around 1 million variants from the ancestral backgrounds: CHB: Han Chinese in Beijing, China; JPT: Japanese in Tokyo, Japan; CHS: Southern Han Chinese; CDX: Chinese Dai in Xishuanagbanna, China; and KHV: Kinh in Ho Chi Minh City, Vietna. The result of this study is shown in **Figure** 4.

To streamline reproducibility we processed this dataset using the popular tutorial from the Laboratory of Complex Trait Genomics (Kamatani Lab) in the Deparment of Computational Biology and Medical Sciences at the Univerty of Tokyo (https://github.com/Cloufield/GWASTutorial). The data was processed in preparation for a typical GWAS study: we selected only autosomal variants, split multi-allelic variants, variants were normalized, remove duplicated variants, selected only SNP (ATCG), selected 2% rare SNPs (`plink -mac 2 -max-maf 0.01 -thin 0.02`, selected 15% common SNPs (`plink -maf 0.01 -thin 0.15`), converted to plink bed format and merged to a single file, and randomly added some missing data points. A prepared version of this processed data is also available from the authors repository in zipped Plink format. Since this public data set (1KG 3v5) as no shared disease phenotype, we followed the same resource to simulate the phenotype file. The simulation parameters were: number of simulation replicate(s) = 1 (Default = 1), heritability of liability = 0.8 (Default = 0.1), disease prevalence = 0.5 (Default = 0.1), number of cases = 250, and number of controls = 254. The QC steps included running `-r2` with the following filters: `-ld-window: 10`, `-ld-window-kb: 1000`,

Click to BUY NOW! PDF-XChange Editor www.pdf-xchange.com
Click to BUY NOW! PDF-XChange Editor www.pdf-xchange.com

`-ld-window-r2: 0.2`, and `-r2 to plink_results.ld`. We then included `-geno 0.02`, `-hwe 1e-6`, `-mind 0.02`. High-LD regions were flagged (`high-ld-hg19.txt`) and pruning was done with `-indep-pairwise 500 50 0.2`. For subject kinship, KING-cutoff 0.0884 was followed by projection (related and unrelated samples).

We performed the single-variant GWAS as a logistic regression with firth correction for a simulated binary trait under the additive mode, with `-maf 0.01`, five PCs, and the simulated phenotypes. Next, we used the default protein pathway gene sets from ProteoMCLustR (https://github.com/DylanLawless/ProteoMCLustR). This consists of a pre-computer set of pathways after cluster the full human genome set of STRINGdb with PPI evidence confidence score 0.7 and contains 941 distinct pathways. We mapped these pathways ID with the variant coordinates (bim file) using the gene coordinates available for this genome build from ensembl biomart `mart_export_GRCh37p13.txt` (https://grch37.ensembl.org/biomart/martview/) for the dataset: human genes (GRCh37.p13), with attributes: Gene stable ID, Gene start (bp), Gene end (bp), Chromosome/scaffold name, Gene name. Our repository also includes `mart_export_GRCh38p14.txt` from http://mart.ensembl.org/biomart/martview/.

We then performed the VSAT with pathway-level variant collapse by running SKAT-O with the `set_ID` assigned to these ProteoMCLustR pathways (e.g. pathway 1,...,n). After QC in GWAS this cohort contained 500 samples and in VSAT we had 945 sets with 1224104 total SNPs. We saved the results from both the Plink single-variant GWAS and the pathway-level SKAT-O. Since this is not a disease cohort and the phenotype is simulated, we automatically increased the association strength of the top VSAT set to provide an enrichment signal. This result is similar to what we have observed in other studies of this size but which do not have public genetic data. Running Archipelago simply requires providing two such inputs (i.e. VSAT results, GWAS results) and any custom theme settings: `archipelago_plot(df_pathway_sim, df_snp_id)`.

The enriched variant signals displayed in **Figure 4** were assessed based on their allele frequency. For the GWAS, the allele frequency of enriched variants ($n = 67$) was: mean = 0.333 , median = 0.315 , min = 0.133 , max = 0.49 which demonstrates that these individual SNP associations were due to highly common variants. For the top VSAT hit (set_ID 532), there were 261 variants. The allele frequency of variants was: mean = 0.151 , median = 0.121 , min = 0.011 , max = 0.478. This demonstrates the scenarios where rare variants within a collapsed set can have a different association than the GWAS region, although this is not always true.

PDF-XChange Editor
Click to BUY NOW!
www.pdf-xchange.com

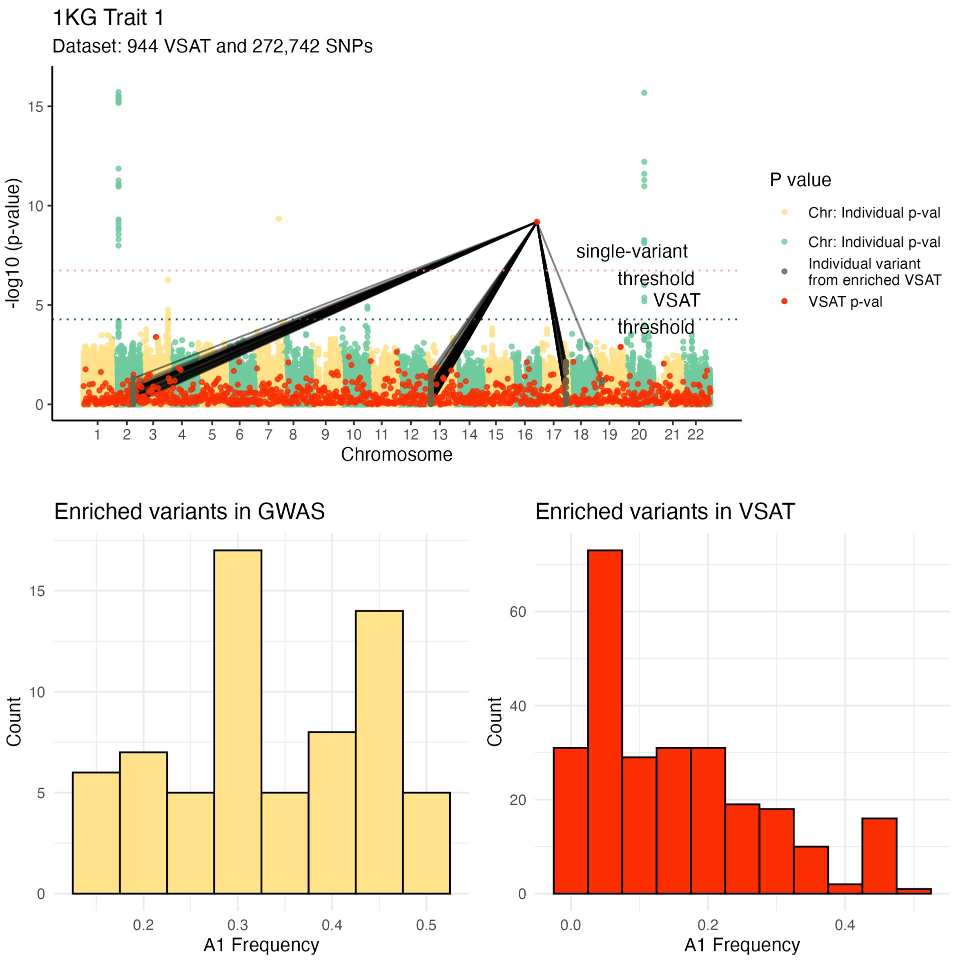

Figure S1: **Allele frequency distributions and signal architecture in the 1KG simulation.** Top: Archipelago visualisation of SNP-level GWAS and pathway-level VSAT results for the simulated binary trait in the 1000 Genomes East Asian cohort. Bottom: Frequency distributions for enriched variants. GWAS-enriched SNPs had a higher mean allele frequency (mean = 0.33) than the top pathway signal (set_ID 532, mean = 0.15), highlighting the complementary resolution of common and rare variant associations.

Click to BUY NOW! PDF-XChange Editor www.pdf-xchange.com

Click to BUY NOW! PDF-XChange Editor www.pdf-xchange.com

13.2.2 Validation method in Pan-UK Biobank using GWAS and gene-level DeepRVAT

To evaluate performance on large-scale data, we applied the Archipelago method to the quantitative UK Biobank trait *platelet distribution width* (phenocode 30110), using two layers: the single-variant level GWAS performed by Karczewski et al. (27) and the gene-level RVAT (which is a VSAT focusing on rare variants) performed in DeepRVAT by Clarke et al. (28).

We retrieved GWAS summary statistics from the Pan-UK Biobank resource (27), comprising 29 million SNPs across 472,000 participants. The gene-level VSAT summary statistics were obtained from the DeepRVAT dataset (6), a published resource accompanying the DeepRVAT method (28). DeepRVAT integrates functional annotations via deep set networks to estimate trait-agnostic gene impairment scores. It tested 3.49 million gene-trait pairs across 97 traits using UK Biobank whole-exome sequencing (WES) data.

To align SNP-level GWAS and gene-level RVAT results, we mapped common variants to protein-coding genes using Ensembl GRCh38 genomic coordinates. The harmonised layers used a shared `set_ID`, defined as the gene ID. For genes without overlapping SNPs ($n = 7$), we assigned the first nearest proxy SNP. Most large-scale studies report summary statistics at a single resolution, so comprehensive overlap between independent GWAS and RVAT datasets remains uncommon. In this analysis, prominent common variant GWAS signals were complemented by additional, independent gene-level RVAT associations whose contributing rare variants were not directly represented in the GWAS layer.

For tractability in plotting, we automatically downsampled the 35,968 gene-level RVAT results by using a ramped log-scale weighting based on $-\log_{10}(p)$. This prioritised moderately significant genes while reducing visual clutter from uniformly null signals, yielding a focused subset of 396 genes for plotting. Data were trimmed at P $<$ 1e-75 for visibility due to large outliers. For easier reproducibility, we also included a randomly downsampled GWAS using 1 in 200 of the 29 million SNPs (yielding $\sim$145,000 variants). Had summary statistics been publicly available at the rare variant level we would also expect additional mapping between the two layers.

13.2.3 Validation method in UKBB WGS UTR collapsing PheWAS

We integrated two layers for a binary phenotype: SNP-level GWAS from the Pan-UKBB resource (27) and gene-level UTR collapsing results from the UKBB WGS

PDF-XChange Editor
Click to BUY NOW!
www.pdf-xchange.com
PDF-XChange Editor
Click to BUY NOW!
www.pdf-xchange.com

PheWAS (8). Here, The UK Biobank Whole-Genome Sequencing Consortium et al. (8) report in Supplementary Table 16 (sheet "Binary") for the 5' UTR, 3' UTR and 5'+3' UTR models. We provided this dataset as the input for the Archipelago VSAT layer. For GWAS, as previously discussed, we mapped variants to protein-coding genes using Ensembl GRCh38 coordinates. We set the VSAT significance threshold based on the original study's guidance for PheWAS ($P \leq 1 \times 10^{-8}$) The UK Biobank Whole-Genome Sequencing Consortium et al. (8) which is equivalent to 5 million tests with Archipelago's default for Bonferroni correction. Archipelago then visualised the aligned layers and linked set-level UTR associations to the gene-based `set_ID` GWAS variants at the *HBB* locus.

13.3 Figure layers

Figures S2a, **S2b**, and **S2c** contain the the same dataset as **Figure 1** however, each version illustrates the decreasing levels of clarification information to demonstrate the layers of annotation. **Figure S2a** shows the original plot without the figure legend. **Figure S2b** drops the edge highlights for the significantly enriched VSAT to reveal all connections, which can be difficult to read in high density plots. **Figure S2c** next drops the individual variant P value highlighting.

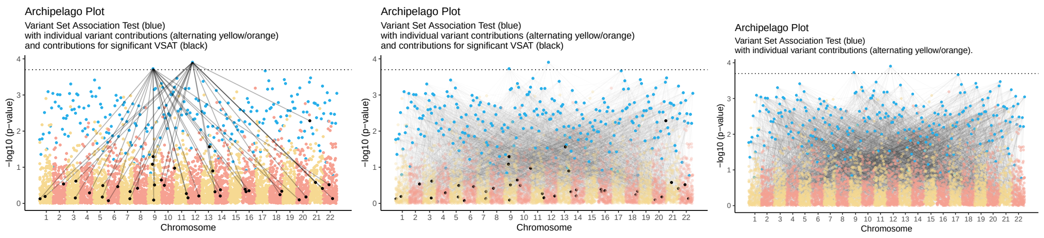

(a) (b) (c)

Figure S2: Illustration of information layers used to reduce complexity.

13.4 Variant set edge colour

Figure S3 adds an additional layer of information to that seen in **Figure S2a** by adding two colours for the significantly enriched variant sets which is useful for when there are multiple enriched variant sets but otherwise may be distracting.

PDF-XChange Editor Click to BUY NOW! www.pdf-xchange.com

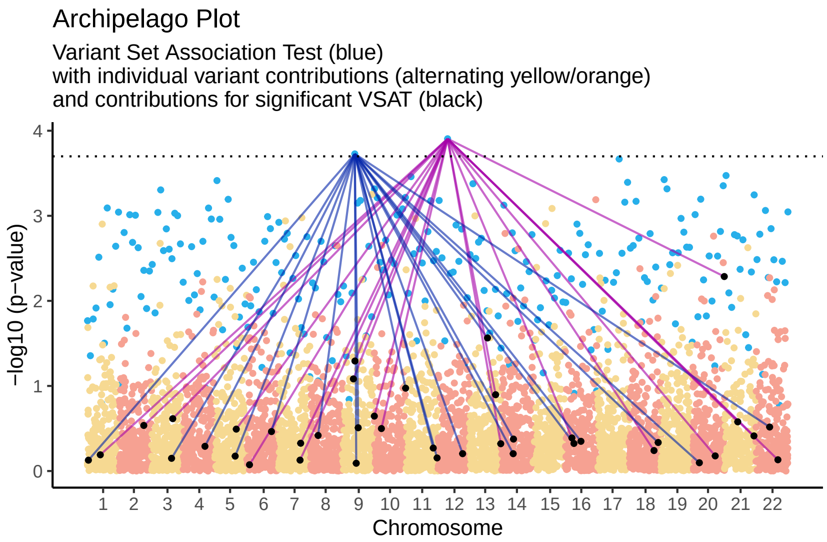

Figure S3: Colored VSAT edges to indicate separate variant sets from two significantly enriched VSAT P values.

13.5 Sparse plots

We demonstrate a set of sparse plots in **Figures** S4a, S4b, and S4c. These smaller dataset examples use synthetic data to represent 500 qualifying variants, and VSAT of 20 genes/variants per variant set (25 VSAT P values). **Figure** S4a illustrates a small variant set of 500 individual variants in 25 variant sets. **Figure** S4b and **Figure** S4c continue by dropping information layers sequentially as previously described in the dense plot examples.

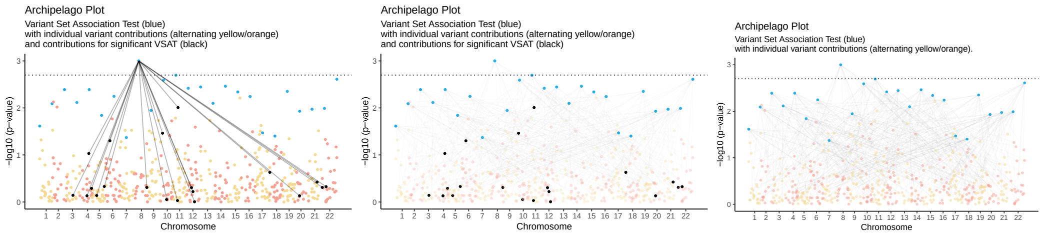

(a) (b) (c)

Figure S4: Illustration of information layers used to reduce complexity in a sparse dataset.

13.6 R package user settings

The R package (logo in **Figure** S9) user settings include:

```r
# Install
install.packages("~/archipelago_0.0.0.9000.tar.gz", repos =
    ↪ NULL, type = "source")

# Load
library(archipelago)

# Load test data for df1 and df2
data("vsat_pval")
data("variant_pval")

# Import user data
df1 <- read.csv(file="../data/vsat_pval.txt")
df2 <- read.csv(file="../data/variant_pval.txt")

# Use default settings
archipelago_plot(df1, df2)

# 8 colour themes:
# 'retro', 'metro', 'summer', 'messenger', 'sunset', 'alice',
    ↪ 'yawn', 'lawless'
output_path = "../output/archipelago_plot_custom_color.pdf"
archipelago_plot(df1,
                 df2,
                 color_theme = 'alice',
                 output_path = output_path)

# Custom everything
color_labels <- c("Label_1", "Label_2" ,"Label_3","Label_4")
custom_colors = c("#9abfd8", "#cac1f3", "#371c4b", "#2a5b7f")
    ↪ # alice theme colors
plot_title <- "Title"
plot_subtitle <- "Subtitle"
crit_val_VSAT = .05/300 # P value threshold line
point_size = .5 # geom_point size
output_path = "../output/archipelago_plot_custom_everything.
    ↪ pdf"
better_space = "TRUE"
```

PDF-XChange Editor
Click to BUY NOW!
www.pdf-xchange.com
PDF-XChange Editor
Click to BUY NOW!
www.pdf-xchange.com

```
archipelago_plot <- function(df1, df2,
                             plot_title = "Archipelago␣Plot",
                             add_title = FALSE,
                             plot_subtitle = "Variant␣Set␣
                                ↪ Association␣Test\nwith␣
                                ↪ individual␣variant␣
                                ↪ contributions\nand␣
                                ↪ contributions␣for␣
                                ↪ significant␣VSAT",
                             add_subtitle = FALSE,
                             chr_ticks = TRUE,
                             show_legend = TRUE,
                             color_theme = NULL,
                             custom_colors = NULL,
                             color_labels = c("Chr:␣Individual
                                ↪ ␣p-val", "Chr:␣Individual␣p-
                                ↪ val" ,"Individual␣variant\
                                ↪ nfrom␣enriched␣VSAT","VSAT␣p
                                ↪ -val"),
                             crit_val_VSAT = NULL,
                             crit_val_single_variant = NULL,
                             point_size = 1,
                             point_size_large = 1,
                             fig_width = 8,
                             fig_height = 4,
                             raw_fig_width = 8,
                             raw_fig_height = 4,
                             output_path = "archipelago_plot",
                             output_raw = "archipelago_vsat_
                                ↪ raw_plot",
                             file_type = "png",
                             alpha_point = 1,
                             alpha_seg = 0.3,
                             better_space = FALSE,
                             legend_position = "right"
                             )


# Print every color theme plot for manual
# List of color themes
```

Click to BUY NOW! PDF-XChange Editor www.pdf-xchange.com

Click to BUY NOW! PDF-XChange Editor www.pdf-xchange.com

```
color_themes <- c('retro', 'metro', 'summer', 'messenger', '
    ↪ sunset', 'alice', 'yawn', 'lawless')

# Loop over each color theme
for (color_theme in color_themes) {
  # Define output path
  output_path <- paste0("../output/archipelago_plot_", color_
     ↪ theme, ".pdf")
  # Generate and save plot
  archipelago_plot(df1,
                   df2,
                   color_theme = color_theme,
                   output_path = output_path,
                   show_legend = FALSE)
}
```

13.7 Protocol Summary

The Archipelago Plot protocol takes as input two datasets containing variant-set association testing (VSAT) P values and single variant P values. It first orders the variant P values by chromosome and genomic coordinate, then averages the coordinates within each variant set. The protocol subsequently normalizes the VSAT x-axis distribution (optional) to avoid center clustering in dense datasets, prioritizing VSAT P values in case of overlaps. It can optionally map the VSAT position to each variant from the set. The protocol then creates a ggplot visualisation, which includes an option to highlight individual variant contributions from significant VSATs.

13.8 Protocol Formal Definition

The formal definition of the Archipelago Plot Protocol comprises the following steps: **Data Preparation**: The data, including variant-set association testing (VSAT) P values and single variant P values, is sorted and averaged within each variant set. **Normalization**: Optionally, the VSAT x-axis distribution is normalized to avoid center clustering in dense datasets. Ranking is done to prioritize VSAT P values in the event of overlaps. **Mapping**: Optionally, the position of each VSAT is mapped to each variant from the set. **Visualisation**: A plot is created using ggplot2, which includes options to add a title, subtitle, chromosome ticks, a legend, and custom

Click to BUY NOW! PDF-XChange Editor www.pdf-xchange.com

Click to BUY NOW! PDF-XChange Editor www.pdf-xchange.com

colours. **Highlighting**: The plot can be made to highlight individual variant contributions from significant VSATs. **Output**: The plot is saved to a specified path and returned by the function.

13.9 Protocol Algorithm

The core of the algorithm can be broken down into the following steps: **Pre-processing**: Load required libraries (ggplot2 and dplyr). Determine the critical value if it's not provided. Data ordering and coordinate calculation: Sort the single variant P values by chromosome and genomic coordinate, and create an index to order the chromosomes. Calculate the position for each data point, essentially ordering the chromosomes end to end, and create mid-points for each chromosome for labeling. **Merging and preparation**: Merge the VSAT P values with the single variant P values, then calculate the sum and average position of each variant set group. Replace missing positions with the average position of their variant set. **Normalization**: If chosen, normalize the VSAT x-axis distribution. This is done by sorting the variant set P values by their position, then assigning evenly spaced numbers across the range of all position values. **Data colouring and splitting**: Assign colours to the different groups in the data and split the data into variant sets and individual variants. **Highlighting**: Define a condition to highlight individual variant contributions from significant VSATs and apply it to the data. **Plotting**: Create the plot with ggplot2, add colour scaling, labels, theme, and optionally title, subtitle, and legend. **Finalization**: Save the plot to a specified output path and return the plot from the function.

The R code is relatively easy to modify or rewrite. Alternative versions could be written based on the following algorithm:

```
Input: D1 (VSAT P values), D2 (single variant P values)
Output: Archipelago Plot

Begin
  1. Load required libraries.
  2. Determine critical value if not provided.
  3. Sort D2 by chromosome and genomic coordinate, create
     ↪ index.
  4. Calculate position for each variant in D2, create
     ↪ chromosome mid-points.
  5. Merge D1 and D2, calculate sum and average position of
```

PDF-XChange Editor
Click to BUY NOW!
www.pdf-xchange.com

```
    ↪ each variant set.
  6. Replace NA positions with the average position of their
    ↪ variant set.
  7. Normalize x-axis distribution of D1 (optional).
  8. Assign colours to data groups, split data into variant
    ↪ sets and individual variants.
  9. Highlight individual variant contributions from
    ↪ significant VSATs.
  10. Create ggplot, add scaling, labels, theme.
  11. Optionally add title, subtitle, legend.
  12. Save and return plot.
End
```

13.10 Customisation

Figure S5 shows an example of customised colours. Figure S6 shows all customisable elements. Figure S7 - S8 shows all 16 colour themes.

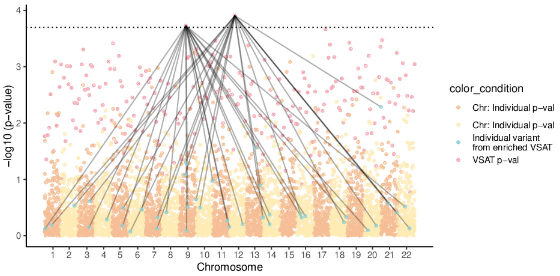

Figure S5: Custom colours specified by hex values, colour names, etc.

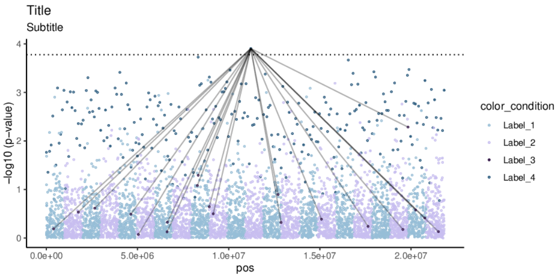

Figure S6: Custom title, subtitle, colours, colour labels, critical threshold line, genomic coordinate, show title and subtitle, and show legend.

PDF-XChange Editor
Click to BUY NOW!
www.pdf-xchange.com
PDF-XChange Editor
Click to BUY NOW!
www.pdf-xchange.com

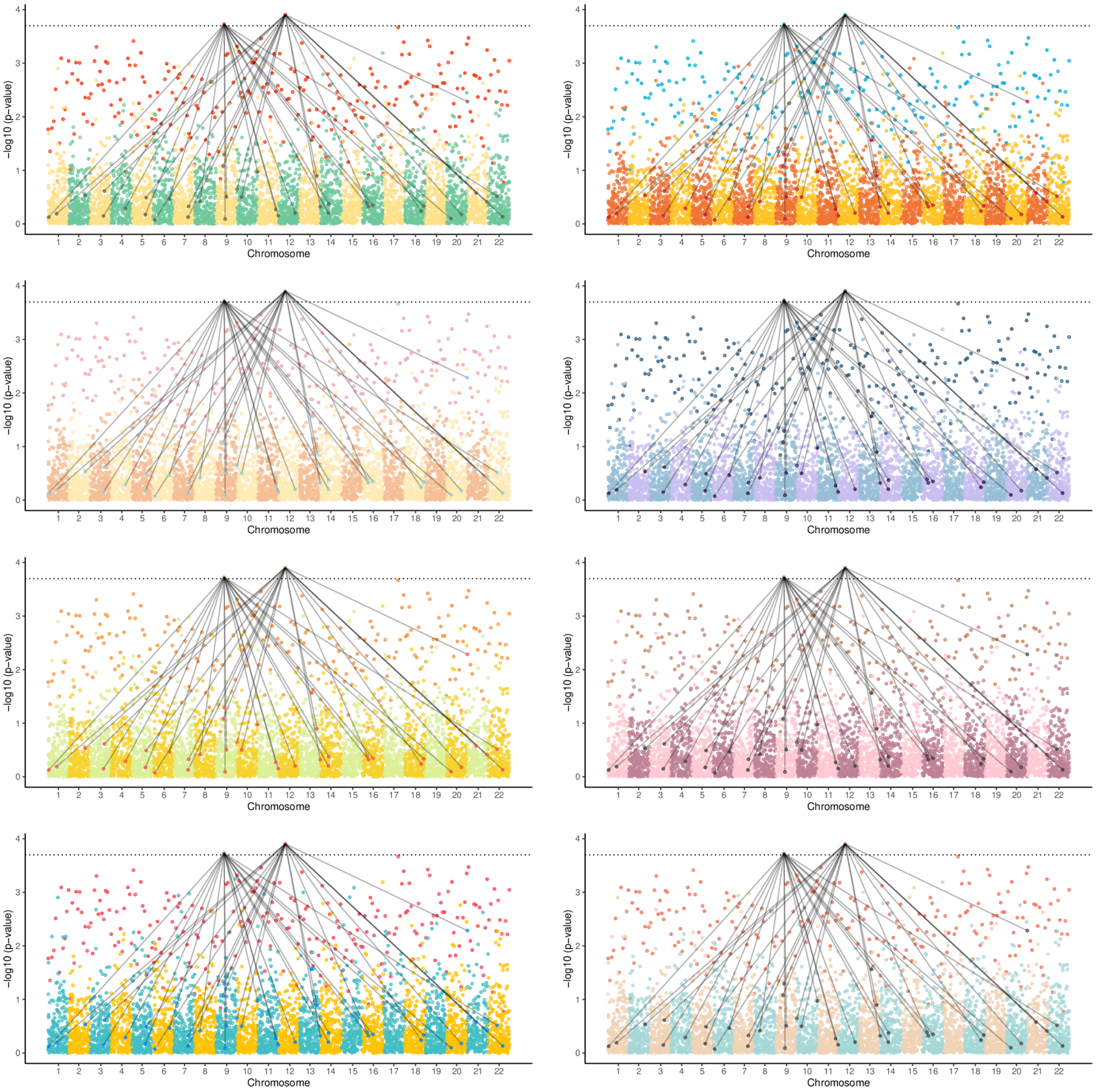

Figure S7: Color themes left to right: 'retro', 'metro', 'alice', 'buckley', 'summer', 'romance', 'messenger', 'meme'.

PDF-XChange Editor Click to BUY NOW! www.pdf-xchange.com

PDF-XChange Editor Click to BUY NOW! www.pdf-xchange.com

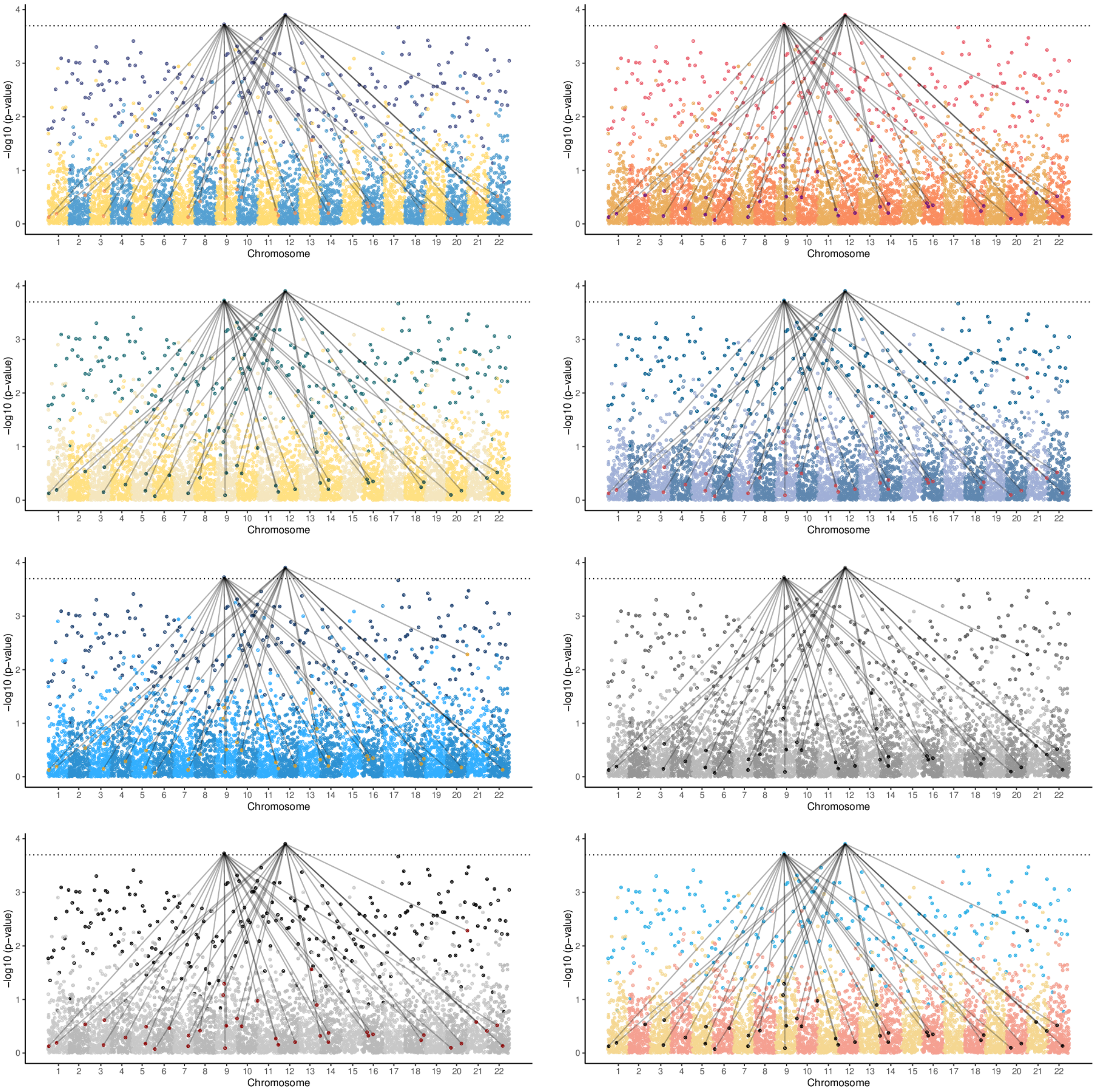

Figure S8: Color themes left to right: ‘pagliacci’, ‘sunset’, ‘ambush’, ‘saiko’, ‘sunra’, ‘yawn’, ‘caliber’, ‘lawless’.

PDF-XChange Editor
Click to BUY NOW!
www.pdf-xchange.com

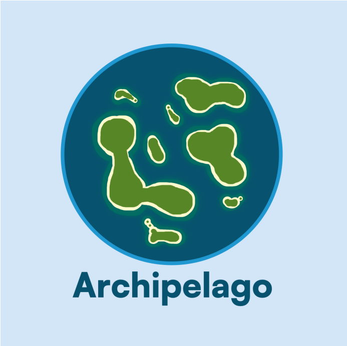

Figure S9: Archipelago plot logo.
